# Direct tracking of reverse-transcriptase speed and template sensitivity: implications for sequencing and analysis of long RNA molecules

**DOI:** 10.1093/nar/gkac518

**Published:** 2022-06-17

**Authors:** Li-Tao Guo, Sara Olson, Shivali Patel, Brenton R Graveley, Anna Marie Pyle

**Affiliations:** Department of Molecular, Cellular, and Developmental Biology, Yale University, New Haven, CT 06520, USA; Department of Genetics and Genome Sciences, Institute for Systems Genomics, UConn Health, Farmington, CT 06030-6403, USA; Department of Molecular Biophysics and Biochemistry, Yale University, New Haven, CT 06520, USA; Department of Genetics and Genome Sciences, Institute for Systems Genomics, UConn Health, Farmington, CT 06030-6403, USA; Department of Molecular, Cellular, and Developmental Biology, Yale University, New Haven, CT 06520, USA; Howard Hughes Medical Institute, Chevy Chase, MD 20815, USA; Department of Chemistry, Yale University, New Haven, CT 06520, USA

## Abstract

Although reverse-transcriptase (RT) enzymes are critical reagents for research and biotechnology, their mechanical properties are not well understood. In particular, we know little about their relative speed and response to structural obstacles in the template. Commercial retroviral RTs stop at many positions along mixed sequence templates, resulting in truncated cDNA products that complicate downstream analysis. By contrast, group II intron-encoded RTs appear to copy long RNAs with high processivity and minimal stops. However, their speed, consistency and pausing behavior have not been explored. Here, we analyze RT velocity as the enzyme moves through heterogeneous sequences and structures that are embedded within a long noncoding RNA transcript. We observe that heterogeneities in the template are highly disruptive to primer extension by retroviral RTs. However, sequence composition and template structure have negligible effects on behavior of group II intron RTs, such as MarathonRT (MRT). Indeed, MRT copies long RNAs in a single pass, and displays synchronized primer extension at a constant speed of 25 nt/sec. In addition, it passes through stable RNA structural motifs without perturbation of velocity. Taken together, the results demonstrate that consistent, robust translocative behavior is a hallmark of group II intron-encoded RTs, some of which operate at high velocity.

## INTRODUCTION

Accurate, end-to-end reverse transcription is required for faithful copying of RNA into cDNA molecules. Despite the critical importance of this reaction in biotechnology and medicine, the speed and behavior of reverse transcriptase (RT) enzymes on mixed sequence RNA templates have not been extensively characterized, particularly for new families of RT enzyme with altered properties, such as enhanced processivity. Conventional RT enzymes (such as the SuperScript series) are primarily derived from retroviral RTs, and these tend to pause or stop upon encountering certain types of RNA sequences, such as repeats (1), and when confronted with stable secondary structures, such as RNA hairpins (2). In these cases, the reported duration of pausing is not uniform, as it can range from seconds to minutes (3). This behavior can confound the interpretation of sequencing data and it can prevent analysis of long templates, or templates containing short regions of stable RNA structure.

In addition to the challenges posed by the diversity of RNA templates, the actual speed along an RNA template at which RT enzymes move and copy RNA are not well known. However, in the few instances in which the RT speed has been characterized, it can be context-dependent. For example, the R2 RT has a maximal speed of 29 ± 9 nt/s on poly(rA) templates, but the speed reduces to 7 nt/s on templates with heterogeneous sequences (4). In that case, apparent pausing events cause the accumulation of truncation products throughout the template. Similar behavior has been reported for AMV (avian myeloblastosis virus) RT (4). Other factors known to impact RT velocity and efficiency include instability of the protein enzyme itself (4). Given the diversity of RNA transcripts that are routinely sequenced with RT enzymes, and the varied types of experimental applications, it is important to develop a clear understanding of the kinetic properties and limitations of these enzymes. Here we analyze the template-dependent speed of RT enzymes, focusing on group II intron-encoded RTs. We examine their responses to barriers in the RNA template, and we compare these properties to a conventional retroviral RT enzyme.

MarathonRT (MRT) is a structurally characterized RT that is encoded within a eubacterial group II intron (5), where it is required for reverse-transcribing intron RNA into DNA during retrohoming (6). Group II introns are self-splicing ribozymes (7,8) with compact, tightly folded structures. To address the challenges presented by this stable template, group II introns have evolved specialized RT enzymes capable of disrupting the secondary and tertiary structures within the intron core, enabling them to copy and propagate themselves as retroelements. Group II intron RTs have acquired specialized structural elements that confer exceptionally high processivity and which enable these RTs to unwind stable RNA structures in their path (9,10). The structure and sequence of group II intron-encoded RTs are distinct from retroviral RTs, having more in common with viral RNA-dependent RNA polymerases (5,11).

In this study, we report the length-dependent velocity and homogeneity of MRT and other RT enzymes in the presence of diverse heterogeneous templates, some of which have been engineered to contain stable structural elements that explicitly test the ‘power’ of the enzyme to pass through stable barriers. We show that MRT carries out synchronized, single-pass primer extension of highly structured templates at a constant speed of 25 nt/s, regardless of position along the RNA template, and it can pass through a diversity of stable RNA substructures without pausing or even slowing down. We find that a related group II intron RT can also traverse stable barriers within the templates, but it has a different intrinsic velocity. This robust behavior of group II intron RTs contrasts with that of commercially optimized retroviral RNA enzymes, which have low processivity, and are blocked upon encounter with stable RNA template structures.

## MATERIALS AND METHODS

### The RT enzymes used in this work

MRT is a group II intron encoded RT derived from *Eubacterium rectale* (5). It was overexpressed and purified to homogeneity as described previously (12). TGIRT, a thermostable group II intron encoded RT derived from *Geobacillus stearothermophilus* (13), was purchased from InGex.com. SuperScript IV is a highly optimized RT derived from Moloney Mouse Leukemia Virus (MMLV RT), purchased from Thermo Fisher. The optimal reaction temperatures for MRT, TGIRT and SuperScript IV are 42°C, 60°C and 55°C respectively. Since the definition of enzyme unit varies for each RT, we provided molar concentration for MRT and TGIRT in the assays. For SSIV, molar concentration is not available, therefore we use the manufacturer suggested enzyme units for each assay.

### Reverse transcription assays

The basic RNA template used for this assay is the HOTAIR lincRNA (2148 nt), which was prepared by *in vitro* transcription (14). The 5′-end of the RT primer (Supplementary Table S1) was labeled with either ^32^P using T4 polynucleotide kinase or with a FAM fluorophore during DNA synthesis. To anneal the primer to HOTAIR RNA, 2 μl of 1 μM primer and 4 μl of 1 μM HOTAIR RNA were mixed, heated to 95°C for 45 seconds, and then cooled down slowly to room temperature. To prepare the reactions, 500 nM MRT, 100 units SSIV or 500 nM TGIRT were mixed with the annealed primer-template, reaction buffer and purified water (Millipore) to make a reaction volume of 19 μl. After pre-incubation of the reaction mixture at 42°C for 1 min to allow the RT enzymes to bind to the primer-template, 1μl dNTP mix (10 mM each, NEB) was added to initiate the reaction (for a 20 μl total reaction volume). The reaction buffer for MRT contains 50 mM Tris–HCl pH 8.3, 200 mM KCl, 2 mM MgCl_2_, 5 mM DTT and 20% glycerol, as previously optimized for MRT (12). For SSIV and TGIRT, reaction buffers and primer extension reaction temperatures (55°C and 60°C respectively), were employed as directed in the manufacturer instructions. For time course experiments, reactions were incubated for the specified reaction times described in the text and then stopped by mixing with 4 μl 10% SDS. For reverse transcription using the HOTAIR templates containing designed RNA structures, reactions were incubated for 5 min for MRT and SSIV and 10 min for TGIRT due to its slower intrinsic velocity. The cDNA products were treated with 300 mM NaOH at 95°C for 5 min to hydrolyze the RNA templates, and then subjected to electrophoresis on a 5% denaturing urea (7 M) polyacrylamide gel. The ^32^P labeled gel was dried and visualized using a Typhoon FLA 9500 scanner (GE Healthcare), and the FAM-labeled gel was directly scanned by the Typhoon FLA 9500 scanner under fluorescence channel. Resulting data were analyzed and quantified using QuantityOne software (Bio-Rad).

### Single-cycle reverse transcription assay

HOTAIR RNA and 5′-FAM labeled RT primer (Supplementary Table S1) were used as the substrate in the single-cycle reverse transcription reactions. To determine the optimal trap for the single-cycle reaction, various molecules were tested, including (1) RNA1 and (2) RNA2, two 50-nt RNA oligos derived from HOTAIR, (3) ssDNA containing a 2′-3′-dideoxycytidine at the 3′-end, (4) RNA1 annealed with unlabeled RT primer, (5) heparin and (6) HOTAIR RNA annealed with unlabeled RT primer (Supplementary Table S1).

The single-cycle reaction procedure was carried out as described previously (10). In brief, 100 nM HOTAIR was annealed with FAM-labeled RT primer in 1:1 ratio and heated to 95°C for 1 min, followed by cooling on ice for 10 min. The annealed primer–template was then incubated with 400 nM MRT in a reaction buffer (50 mM Tris–HCl pH 8.3, 200 mM KCl, 2 mM MgCl_2_, 5 mM DTT and 20% glycerol) at room temperature for 10 min and then 42°C for 1 min. Subsequently the dNTPs, in combination with the respective trap molecule, were added to initiate the single-cycle reaction. The final concentration for the dNTPs was 0.5 mM, and the final concentration for each of the traps was described in the legend (Supplementary Figure S6). Single-cycle primer extension reactions were incubated at 42°C for 1 min and stopped by SDS. For the time-course experiment, RNA1 at a final concentration of 6 μM was used as the trap, and the reactions were carried out for 10–90 s as specified in the text. Reactions were stopped with the addition of SDS and analyzed by denaturing polyacrylamide gel as described.

### Cloning of stable RNA structural motifs into the HOTAIR sequence

The RNA structural motifs were inserted into the sequence of HOTAIR RNA between position 1512 and 1513, between Domain 3 and 4 (14). The DNA fragments encoding these RNA structures were inserted into HOTAIR gene using the seamless cloning approach provided by Gibson Assembly (New England Biolabs). See Supplementary Table S2 for the inserted sequences.

### Oxford Nanopore Sequencing Library prep and data analysis

Reverse transcription was done with MRT as described above and cDNA:RNA duplex products were purified by ethanol precipitation. For each sample, ∼100 fmol of purified cDNA:RNA duplex was subject to second strand synthesis using NEBNext Double Stranded cDNA Synthesis Kit (New England Biolabs), following manufacturer's protocol. Subsequently, double stranded DNA products were prepared for Nanopore sequencing using Oxford Nanopore Ligation Sequencing Kit and Native Barcoding according to manufacturer's protocol. A unique barcode was used for each sample. Final libraries were pooled and sequenced on a FLO-MIN106, R9.4.1 flow cell in a MinION device, using MinKNOW software v. 19.12.5 for device control and data collection. Output was base-called using Guppy (v2.2.3, default settings) (15). The resulting fastq files were filtered to remove adapters (internal or end adapters) using Porechop 0.2.4 (default settings, https://github.com/rrwick/Porechop) and aligned to the reference sequence using ngmlr 0.2.7 with the parameter for Oxford Nanopore reads, -x ont (16). Thereafter a custom python script (see supplementary data) was used to determine the cDNA lengths.

### Illumina sequencing and determination of reverse transcription stops

Briefly, reverse transcription was performed with MRT, TGIRT and SSIV at 42°C, 60°C and 55°C for 5, 10 and 5 min, respectively as described. Four RT primers were used to cover the entire length of the GCSL HOTAIR template (Supplementary Table S1). cDNA products were purified using AMPure XP beads by adding 1.2x bead to sample ratio according to manufacturer's protocol. Thereafter the cDNA was 3′ adapter (Supplementary Table S1) ligated using T4 RNA Ligase 1 (NEB) by mixing 8 μl of cDNA with 0.2 μl of 50 μM 3′ adaptor, 1 μl of 10 mM ATP, 2 μl of T4 RNA Ligase buffer, 8 μl of 50% PEG 8000. The mixture was incubated at 25°C for 16 h, followed by enzyme deactivation at 65°C for 15 min. Ligated products were then cleaned with AMPure XP beads by adding 1.2× bead to sample ratio. The resulting products were PCR amplified 5–9 cycles with Q5 DNA polymerase (NEB) using Illumina TruSeq forward primer and indexed reverse primers (NEBNext multiplex oligos), with cycles of 98°C, 10 s; 62°C, 30 s; 72°C, 60 s. Multiplexed sequencing libraries were pooled and sequenced using a NextSeq 500/550 platform using a 150 mid output kit.

FASTQ files were trimmed with Cutadapt (v3.2) to remove Illumina adaptor sequences and aligned to the GCSL template with HISAT2 (v2.1.0). The stop information was extracted with software RTEventsCounter.py (https://github.com/wyppeter/RTEventsCounter) (17). To reduce the inherent bias that is introduced by clustering long libraries on an Illumina Next-Seq flow cell, we utilized band densitometry to calculate the fraction of cDNA extension reactions that stopped prematurely due to the presence of the GCSL (F_stop_). The intensity corresponding to the GCSL stop site and that of longer extension products were calculated I_GCSL_, (red asterisk in Figure 4B–D) and I_readthrough_, respectively. The fraction of cDNA population stopped due to the GCSL structure was defined as

$$\begin{equation*}{{\rm{F}}_{{\rm{stop}}}} = {{\rm{I}}_{{\rm{GCSL}}}}/{\rm{ }}\left( {{{\rm{I}}_{{\rm{readthrough}}}} + {\rm{ }}{{\rm{I}}_{{\rm{GCSL}}}}} \right)\end{equation*}$$

## RESULTS

### Monitoring MarathonRT behavior as a function of time and template position

When RT efficiency is evaluated by users or developers for most applications, the enzyme is incubated with primer-template for a fixed period of time and the relative amount of primer extension is quantified, without considering the diverse physical processes that contribute to efficient primer extension on a given template. This becomes problematic when working on exceptionally long or complex templates, as it is important to know the actual speed and constraints on the polymerase so that one can gauge appropriate reaction times. In addition, most templates contain at least some short regions of stable secondary structure or repeat sequences and it is important to know how and whether these are traversed by the enzyme. Finally, an important metric of efficiency are the changes in speed or processivity that a polymerase displays during the initial, middle, and final stages of a template-copying reaction, as this will affect the number of times each base being copied as a function of position. To evaluate all of these attributes, we developed a method for monitoring the behavior of RT enzymes in real time as they travel along a test template.

**Figure 1.**
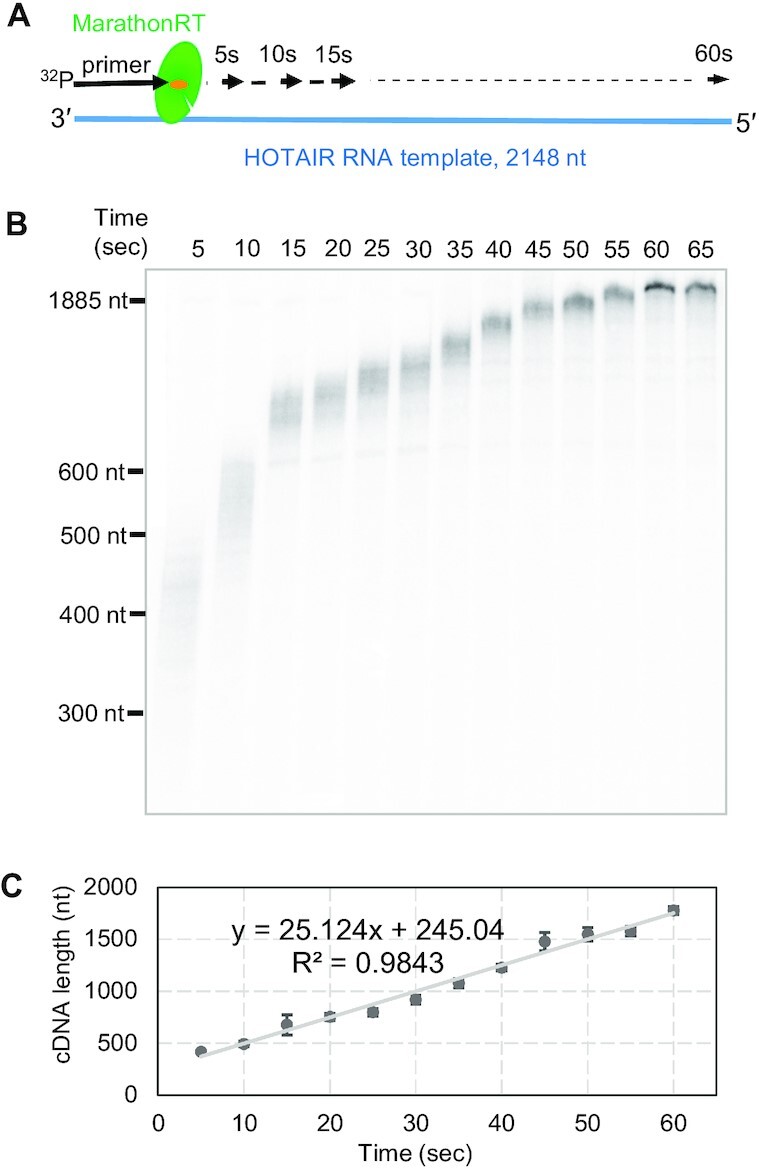
High-resolution time course experiment to monitor the primer extension progress of MRT. (**A**) Overview of the primer extension time course experiment. MRT that was extending the ^32^P-labeled primer along the 2148-nt HOTAIR RNA template was stopped at the indicated reaction times in 5-sec increments until it reached the end of the template. (**B**) Electrophoresis analysis of the cDNA products generated in the time-course experiment on a denaturing polyacrylamide gel. The full-length cDNA is 1883 nt. (**C**) Plotting of cDNA length against reaction time to calculate the velocity for MRT. The cDNA length was estimated by comparing with DNA size markers. The average length for each cDNA sample was calculated based on three replicates. The error bar for each time points represents standard deviation. The velocity was determined to be 25.1 ± 1.1 nt/s.

Specifically, reverse transcription was initiated from a ^32^P- or fluorophore-labeled primer that had been annealed to a long RNA template containing diverse sequences and secondary structures. After incubation of RT enzyme with primer-template, the primer extension reaction was initiated with a rapid pulse of dNTPs. The reaction was stopped at a series of short time intervals (Figure 1A) and the products were evaluated by electrophoresis or nanopore sequencing. The cDNA lengths produced at each reaction time were determined by comparison with DNA size markers. After plotting cDNA length against reaction time, one can gauge the relative synchrony of the RT population, visualize pauses and stops of subpopulations along the path and determine global and local velocity by linear regression. For our studies, it was important to select an RNA template that was long enough for monitoring the progression of extending primers over time, but short enough to be analyzed on a high-resolution polyacrylamide gel. Based on the calculated velocities of other single-chain polymerases, we hypothesized that RT velocity would be lower than 50 nt/sec (4,18). To meet these objectives, we chose a 2148-nt lncRNA known as HOTAIR (HOX transcript antisense RNA) as the template, as this would provide a window of at least 40 seconds for actively monitoring primer extension. In addition to being a long RNA, HOTAIR contains heterogeneities in sequence and structure that might be expected to provide insights into RT response. The HOTAIR secondary structure has been determined (14), it contains several repeat sequences, and its local GC content varies significantly across the template with a gradual decrease from 5′- to 3′-end (Supplementary Figure S1). Primer extension along HOTAIR provides an opportunity to closely examine the behavior of MRT and other RTs under a diverse set of natural conditions.

Given that the time points were necessarily fast in this experiment, it was essential to develop a method for rapidly inactivating the actively translocating enzymes. We tested rapid heating and fast mixing of SDS to the reactions, finally settling on an SDS quench method for terminating active reverse-transcription. To monitor time courses consistently, each reverse transcription reaction was monitored individually at a series of fast reaction times (see Materials and Methods).

Remarkably, time-courses of MRT primer extension revealed a number of unexpected behaviors. First, the population of advancing enzymes was observed to be highly synchronized (Figure 1B), migrating as single large population through time after initiation of reaction. Second, this synchronized population of primer extension complexes was maintained throughout the course of reaction, suggesting that enzyme functional properties are not deteriorating or changing with time (Figure 1B). Perhaps most interesting is that the front of advancing enzymes appears to move at the same speed along the entire template (Figure 1C). Given the synchrony and homogeneity of behavior of this enzyme, it became evident that we could utilize this method to calculate the velocity of MRT, and to evaluate whether the relative speed changes with time or position on the template.

### Determining the velocity of MarathonRT and related enzymes

To determine the velocity of MRT, we plotted the average length of radiolabeled cDNA products as a function of reaction time (Figure 1C), resulting in an overall speed of 25 ± 1.1 nt/s. Remarkably, the velocity plot is perfectly linear throughout the course of reaction, which indicates that MRT moves at constant speed and that activity is not diminished at later stages of the reaction. In addition, the population of extending primers at the front does not diminish as it moves along the template (Supplementary Figure S2), which would otherwise occur with retroviral RTs, caused by frequent premature stops. The end-to-end, unbiased reverse transcription of a long RNA template with MRT has important implications for homogeneity of read depth in RNA sequencing experiments. To examine this phenomenon using an orthogonal method that would enable us to evaluate the resulting cDNA products more quantitatively in downstream nanopore experiments, we repeated the time-resolved extension reaction using primers labeled with 5′-FAM (5′-carboxyfluorescein), resulting in a MRT velocity of 25.4 nt/s (Supplementary Figure S3). This confirms the absolute value for the speed of MRT and shows that 5′-fluorescent labelling of the primer does not affect efficiency of the extension reaction. Again, it is striking that, with both types of labeled primers, linear regression of the resulting velocity plot reveals that the speed of MRT is completely constant across the full length of the template (*r*^2^ > 0.98) (Figure 1C and Supplementary Figure S3).

The constant speed of MRT in this context is particularly significant given that HOTAIR is structurally complex, containing local regions of stable secondary structure and repetitive sequences (14). Most polymerases pause, stall or stutter at template heterogeneities (3,18), and it was therefore remarkable that MRT not only shows minimal stops, but it does not even slow down upon encountering with any of the substructures within the HOTAIR template and there is no decrease in activity after traveling ∼1900 nucleotides along the expanse of HOTAIR. This behavior indicates that MRT can disrupt base pairs and other RNA-RNA interactions within the structure of HOTAIR and that this process of template smoothing never becomes rate-limiting for the reaction, implying that some other step, such as translocation, is rate-limiting. In addition, the results indicate that the enzyme is stable under the reaction conditions used for the study.

This behavior is in sharp contrast with the apparent behavior of retroviral RTs, which are obstructed by certain sequences and secondary structures during primer extension, displaying pauses, stops or bursts of dissociation at different types of template heterogeneities (19,20). To directly compare the behavior of MRT with other types of RTs, we first performed a time-course experiment for the commonly-used retroviral RT SuperScript IV (SSIV, derived from MMLV RT) and we observed that the primer extension was dominated by multiple premature stops that prevented most of the extending primers from reaching the end of the template (Supplementary Figure S4). Due to this behavior, and the fact that it is a distributive enzyme that does not function under single-cycle conditions (in which the same enzyme completes cDNA synthesis from end-to-end) (10), it was not possible to estimate the speed of SSIV. We then examined the behavior of another group II intron-encoded RT, TGIRT (thermostable group II intron RT). Time courses of TGIRT primer extension reflect continuous extension that is similar in many ways to that of MRT, although TGIRT proceeds at slower speeds under its optimal reaction conditions (4.4 nt/s, Supplementary Figure S5). These results are consistent with the suggestion that group II intron-encoded RTs have evolved to efficiently disrupt RNA template structures (10,13), although their basal rates of primer extension can differ, with implications for the timeframe needed to copy kilobase-length transcripts.

### Monitoring and profiling MarathonRT velocity with nanopore sequencing

**Figure 2.**
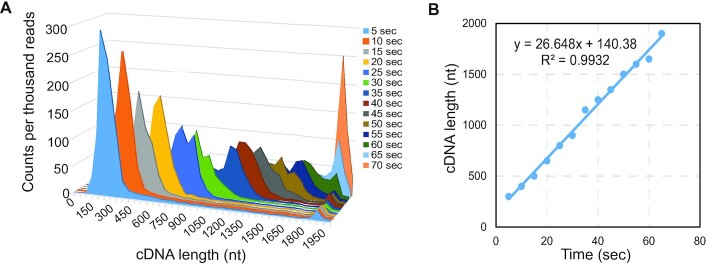
Nanopore sequencing analysis of the cDNA products generated by MRT using HOTAIR template in a time course experiment. (**A**) Length distribution plotting of the sequencing reads that represent the cDNA products generated at different reaction times. (**B**) Plotting of the average cDNA length determined by nanopore sequencing against reaction time, fit to a linear model. MRT velocity was determined as 26.6 nt/s.

While electrophoresis is a rapid method to determine cDNA distributions and velocity, the calculated sizes are limited to resolution on the gels. Therefore, we wanted to use an orthogonal approach to estimate distribution of cDNA products at the reaction time points. Due to the large size of the RNA, we utilized Oxford Nanopore sequencing, a method that allowed us to use a single RT primer for sequencing the various length cDNAs at all time points. While we cannot determine single nucleotide resolution with precision, we were able to sequence individual cDNAs produced at each time point which allowed us to obtain distribution plots for the products (Figure 2A). Upon inspection of the curves, we saw a similar synchronized population of primer extended cDNA products over time. Though the amplitude of the curves decreases over time, this is attributable to biases in nanopore library construction and sequencing. Moreover, while some degree of desynchronization can be happening there is minimal loss of elongating products as the final 70 sec peak amplitude is similar to that of the initial starting product.

To determine elongation velocity from the global nanopore data, we calculated the distribution peak maximums at each time point and subsequently plotted these values against reaction time. Notably the calculated velocity by sequencing, 26.6 nt/s (Figure 2B), matched well with the calculated velocity by electrophoresis, 25.4 nt/s. Overall, utilizing each method confirms the consistency and accuracy of the velocity for MRT.

### Analyzing the synchronized primer extension under single-cycle conditions

During the velocity experiments described above, an excess of MRT enzyme was used to carry out the reactions, which is classically defined as a ‘multiple-cycle reaction condition’. This condition is typical for routine primer extension reactions using reverse transcriptases (12,13). The shortcoming of this experimental condition is that one cannot monitor the speed or product length from a single molecule of enzyme, as the excess RT enables a second molecule of enzyme to bind at premature terminations and continue the primer extension. Multiple-cycle conditions are actually essential for the activity of distributive retroviral RT enzymes (such as the SuperScript series), as they require multiple enzymes to carry out a single copying event (10,12). By contrast, single-cycle conditions are required to determine if the synchronized pattern we observe for MRT is the macroscopic observation of a large group of enzyme molecules that are uniformly and continuously extending the primers, without a pause, stop, dissociation or reassociation event that would disrupt the continuity of primer extension and thus result in desynchronization.

**Figure 3.**
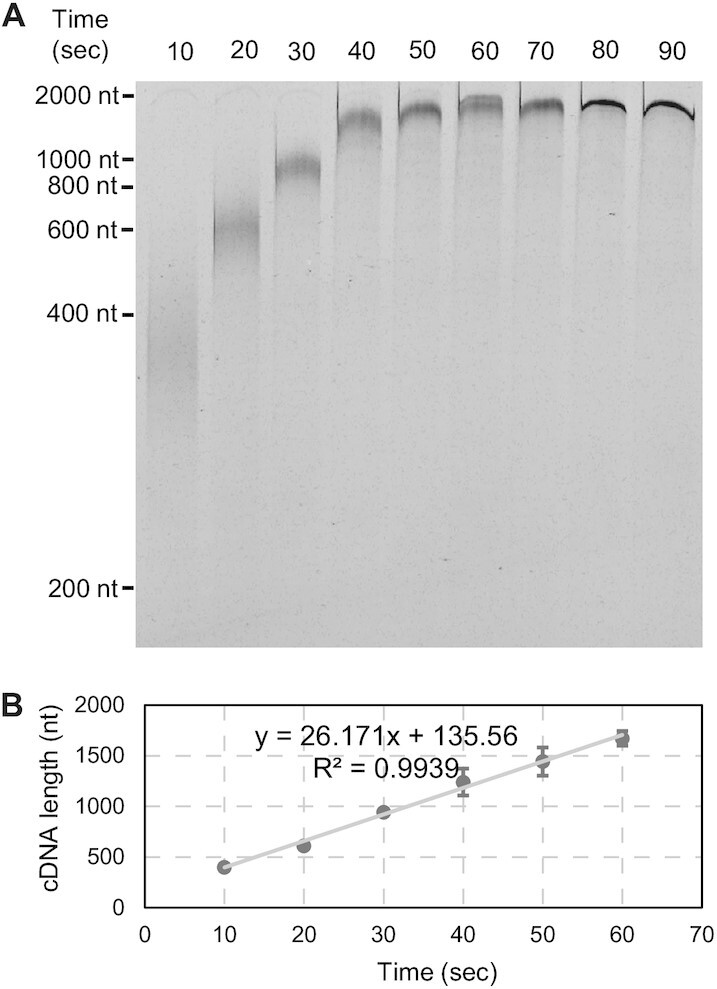
Analyzing primer extension progress by MRT in time course under single-cycle conditions. (**A**) Electrophoresis analysis of time-resolved cDNA products on a denaturing polyacrylamide gel via FAM labeling. (**B**) Plotting of the average length of cDNA against reaction time. The average velocity calculated from the three replicates is 26.2 ± 3.0 nt/s. The error bar for each time points represents standard deviation.

To determine if the velocity we calculated is for a single MRT molecule copying in a single pass, we evaluated the velocity of MRT under single-cycle conditions, in which an excess of trap was added upon initiation of primer extension as described previously (see Materials and Methods) (10). Before initiating the study, we screened a diverse set of traps (see Materials and Methods) and found that a 50-nt RNA oligonucleotide derived from the 3′-end of HOTAIR effectively prevents reassociation of the RT but does not decrease the velocity of MRT. Effectiveness of the trap was established by observing that cDNA products were not detected when trap is preincubated with MRT before adding primer-template duplex and that MRT extends the primer to the end of HOTAIR template in one minute (Supplementary Figure S6, lanes 1 and 2). We then performed a brief time-course experiment to determine the velocity for MRT by using the RNA trap to force the primer extension under single-cycle conditions. The kinetic behavior of primer extension observed under multiple-cycle conditions, including the highly synchronized pattern of primer extension and the constant velocity across the template, was also observed under single-cycle conditions, resulting in a speed of 26 ± 3.0 nt/s (Figure 3). Thus, the synchronized primer extension by MRT results from an ensemble of single-enzyme reactions in which each RNA is copied in a single pass by a single molecule of enzyme. This is consistent with our previous studies showing that MRT has a processivity coefficient of over 30 000 nt (10).

### Systematic analysis of template structures on primer extension

**Figure 4.**
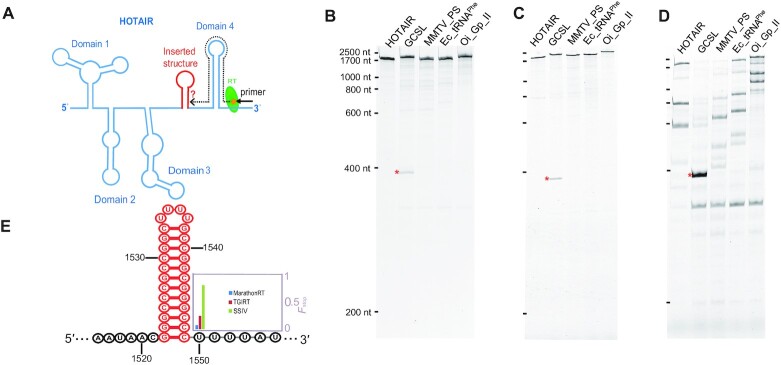
Evaluating the impact of stable RNA structures on the processivity of MRT, TGIRT and SuperScript IV. (**A**) Schematic overview showing each of the stable RNA structures (red) that were inserted at the same position between Domain 3 and 4 of HOTAIR RNA. The RT primer was annealed at the 3′-end of the templates for extension by each of the three RT enzymes. The inserted RNA structures included an ultra-stable 12-bp GC stem loop (GCSL), an MMTV frame-shift site pseudoknot (MMTV_PS), an *E. coli* tRNA^Phe^ (Ec_tRNA^Phe^), and an *O.i*. group II intron (Oi_Gp_II). Electrophoretic analyses of cDNA products generated by (**B**) MRT, (**C**) TGIRT and (**D**) SSIV from wild-type HOTAIR RNA (HOTAIR) and the HOTAIR variants. The prematurely terminated cDNA products induced by the GCSL are denoted by red asterisks. (**E**) Secondary structure of the GCSL stem loop (red), with an insert to display the F_stop_ of 0.08, 0.26 and 0.86 for MRT, TGIRT and SSIV, respectively (see Materials and Methods).

In the previous experiments, we used a relatively structured lncRNA (HOTAIR) as the template. The homogeneity of velocity along the length of this RNA suggested that the group II intron-encoded RTs can readily unwind RNA structures formed within the HOTAIR template. But none of the HOTAIR substructures represent unusually difficult, thermodynamically stable obstacles and so we wanted to provide a more challenging test. To systematically evaluate the ability of RT enzymes to open and copy a diversity of stable RNA substructures, we selected a set of stable RNA secondary and tertiary substructures as tests for the unwinding power of the enzymes. These RNA substructures were then imbedded into HOTAIR RNA to create challenge templates for reverse-transcription (Figure 4A). These RNA substructures include a highly stable 12-bp GC stem loop (GCSL) (Δ*G* = –33.32 kcal/mol), the mouse mammary tumor virus (MMTV) gag-pro shift site pseudoknot (21), *Escherichia coli* tRNA^Phe^, and *Oceanobacillus iheyensis* group II intron (22) (Supplementary Table S2 and, illustrated, Supplementary Figure S7). These structures were inserted between HOTAIR positions 1512 and 1513, which is the boundary that separates HOTAIR Domain 3 from 4, to minimize the impact on the formation of the modularized HOTAIR structure and the substructures themselves. The primer used to reverse-transcribe HOTAIR RNA variants is same as that used for the previous velocity experiments. In this case, RTs examined in this experiment (which include MRT, TGIRT and SSIV) carry out primer extension for ∼370 nucleotides before encountering the inserted RNA structure obstacles.

Overall, in the case of MRT and TGIRT, the inserted RNA structures had minimal influence on processivity, speed or pausing of the enzymes (Figure 4B and C). However, they had a profound impact on SSIV as there are a multitude of stops throughout the various templates, including known stem-loops within the HOTAIR sequence (Figure 4D), which reflects the inherent shortcoming of this retroviral RT. Interestingly, the presence of the GCSL structure caused perturbation of extension by all three enzymes, denoted by red asterisks. For MRT 8% of the extending cDNAs were stopped upon encountering the GCSL, whereas for TGIRT nearly 25% stopped. In the case for SSIV, the presence of the GCSL blocked 86% of extending cDNAs. We calculated the velocity of MRT as it reverse-transcribes along the GCSL template. Of the extending population that can make it past the GCSL, we observe a normal, synchronized pattern of primer extension and the same constant speed observed previously for the WT template (Supplementary Figure S8), suggesting that the GCSL obstacle does not cause MRT to slow down.

### Determining the exact location of the GCSL induced stop

To provide information on the physical mechanism of template smoothing by RT enzymes, we sought to determine the exact site of the RT stop induced by the GCSL structure for each enzyme. To do so, we used a previously developed method that exploits next-generation sequencing to analyze the location of reverse transcription stops on the GCSL HOTAIR template (17). For all three enzymes, a stop was detected at nucleotide position 1550 (Figure 4E), although as described above, each enzyme had a different probability of proceeding past the stem-loop. This stop leads to a truncated cDNA product of 378 nt, which agrees well with the band size observed by electrophoresis as denoted by red asterisks (Figure 4B–D). This nucleotide is positioned exactly at the base of the stem–loop, suggesting that the polymerization active sites of all the three RTs can get very close to the base of the GCSL structure before the complex is destabilized.

## DISCUSSION

Here, we present the first study in which the speed of elongating RT enzymes has been continuously measured along a long, mixed sequence template typical of cellular RNAs, with results that have important implications for our understanding of the mechanism and implementation of these critical enzymes. Understanding the absolute value of RT speed is particularly important when working with group II intron RTs such as MRT and TGIRT because of their extremely high processivity, but it is also important for applications involving the distributive retroviral RTs such as SSIV, which we included in a comprehensive comparative analysis of velocity dynamics. Given that our approach provides positional-dependent information on velocity, it also enabled us to monitor the behavior of RT enzymes when they encounter structural obstacles during elongation. To our knowledge, this is the first time that structurally-defined, stable RNA structural motifs have been systematically placed at specific sites in the path of an elongating RT in order to evaluate the dynamical response of the enzyme before, during and after encounter with the obstacle. Our findings provide unexpected insights into the types of RNA structures that block RT enzymes and, for some of the RTs, they reveal surprisingly robust behavior post-encounter.

### Different motors, different speeds: characteristic velocities of RT enzymes

In this study we show that the highly processive RT enzymes MRT and TGIRT carry out efficient primer extension at constant speeds along mixed sequence templates, although the absolute values of their velocities are different (25 nt/s and 4.4 nt/s, respectively). While both enzymes are derived from group II encoded RTs, differences in the absolute value of their speed may reflect different inherent properties of each enzyme, as they are not identical. They share only 43% sequence identity and 62% sequence similarity. TGIRT is derived from a thermophilic organism, and may therefore have evolved enhanced binding affinity to RNA template-primer to improve thermostability (23). This may require more energy to overcome the strong interaction with template-primer and, consequently, reduce the primer extension velocity. However, TGIRT is not a native RT enzyme, as it is fused to Maltose Binding Protein (MBP) to enhance solubility, which may impact its apparent velocity (13).

Unlike other RT enzymes, which can only be examined under multiple-cycle conditions (10), it is possible to study MRT under single-cycle reaction conditions in which the enzyme cannot rebind after falling off. This enabled us to show that, at least for MRT, individual RT are moving along the entire template length, copying RNA as a homogeneous population of molecules with a uniform velocity. For the first time, this enabled us to compare an RT enzyme with the observed velocity of other single-chain polymerases that are processive under single-cycle conditions. One example is the highly processive DNA polymerase ϕ29, which carries out primer extension on M13 ssDNA at a rate of 23.3 nt/s (1400 nt/min) (24). Other examples include the thermostable *Pfu* and Deep Vent DNA polymerases, whose extension speeds were determined to be 25 and 24 nt/s on M13 ssDNA, respectively, at their optimal extension temperatures of 75°C. Remarkably, MRT is quite comparable in speed to these polymerases and our results suggest that a non-obstructed extension speed of ∼25 nt/sec is some type of ‘sweet spot’ in the efficiency of single-chain polymerases. The universality governing their rate-limiting mechanism merits further exploration.

### Mechanistic implications of constant speed

The observed kinetic behavior of MRT has important mechanistic implications. The constant, unperturbed speed of MRT along mixed sequence templates implies that the enzyme behaves much like a translocative helicase, which strips away obstacles in its path (whether its protein or base-paired RNA/DNA). The constant velocity of the enzyme, regardless of position along the template, also implies that the protein itself does not undergo rate-limiting conformational sampling as it travels or encounters features in the template, and that the active structure is highly stable, which is not surprising given the bioinformatic stability search that was used to discover the enzyme (5). Such properties of MRT are similar to those of the single-chain ϕ29 DNA polymerase, which is also a highly stable protein that catalyzes uniform primer extension that is unperturbed by template heterogeneity (24). During the course of evolution, both enzymes appear to have acquired additional domains that facilitate consistent, processive primer extension (10,25), although these domains differ in apparent structure. The efficient and consistent kinetic behavior of MRT probably stems from the fact that it is a component of a genetic parasite that evolved to function in foreign cellular environments with diverse conditions and template structures. Fitness of mobile elements like group II intron RTs requires that they are self-contained and unperturbed by the host environment.

### Practical implications of RT enzyme speed

The specific velocity of an RT enzyme has important practical implications for its use during primer extension reactions and its application as a biotechnological tool. Our findings demonstrate that, even for the most processive RTs available, one must be cognizant of the inherent enzyme velocity when planning primer extension experiments, particularly when performing reactions on templates that are kilobases in length. This is important because viral RNA genomes, long noncoding RNAs and full-length mRNAs are the focus of intense current research, and many of these transcripts are often very long, exceeding kilobases in length. Given the velocities we have measured, the reaction time for a 10kb template would be at least 7 min for end-to-end coverage by MRT at 42°C and 40 min for TGIRT at 60°C. Furthermore, while most reverse transcription protocols suggest an incubation of 1 hour at the optimal reaction temperature our data suggests one can significantly reduce this time for MRT, which streamlines long, multistep protocols. Additionally, MRT functions under mild reaction conditions, with an optimal reaction temperature of 42ºC for full activity whereas for TGIRT, the optimal reaction temperature is 60ºC. A recent study has shown that RNA is relatively stable at 45ºC (depending on magnesium ion concentration and pH), but the rate of degradation increases by nearly 10-fold at 60ºC (26). Using MRT at 42°C and a shorter incubation period will reduce the time the RNA is exposed to high temperatures and be subject to template breakage. Thus, the speed of a given enzyme and the incubation temperature impacts the feasibility of specific experiments, particularly when working with long RNA molecules.

### Speed bumps and roadblocks: what happens when RT enzymes collide with obstacles?

Naturally-occurring RNAs contain a multitude of mixed nucleotide sequences, including repeat sequences and regions of stable RNA structure. While previous studies suggest that template secondary structures can induce premature RT stops, a systematic characterization of RT response to different types of RNA structural states and obstacles has never been done until now. To address this issue, we designed a series of stable ‘challenge structures’ (ranging from stable RNA stems to entire tertiary structures) and then created a set of ‘test templates’, in which we imbedded each ‘challenge structure’ at the same position in the center of a long RNA template, placing them well in the path of the elongating polymerase (Figure 4A and Supplementary Figure S7). Specifically, the primer binding site was located ∼370 nt away from the challenge structure, enabling us to observe behavior of the elongating RT as it encounters each different type of obstacle. This design consideration is important, as previous studies of RT/template interactions involved the analysis of RNA stems that were incorporated into very short templates, immediately adjacent to the RT initiation site (27). Such a design may confound interpretation of RT behavior by influencing the earliest stages of productive initiation and preventing the enzyme from ‘revving up’ and running unperturbed in continuous elongation mode. Having created our novel series of test templates, we then initiated primer extension with the various families of RT enzyme and compared their relative behaviors.

We observe that engineered template structures have minimal impact on the group II intron encoded RTs (MRT and TGIRT), but they block even the most highly optimized retroviral RT (SSIV). Surprisingly, the obstacle that caused the biggest disruption to the RTs in this study was not the imbedded tRNA or an intact group II intron, but a relatively small stem-loop that is rich in G-C pairs (GCSL). To obtain a better understanding of where the RT enzymes stop relative to the position of the stem loop, we utilized a high-throughput, single nucleotide resolution approach to determine exact stop sites induced by the GSCL for all three RT enzymes (17). The majority of group II intron RT molecules pass through the GCSL and copy it successfully, but a small population is stopped by this substructure. By contrast, almost the entire population of SSIV is blocked by the GCSL. For all stops observed at the GCSL, all three RTs synthesize cDNA right up to the basal position of the GCSL stem before stopping. It is therefore clear that the GCSL loop passes through the entry tunnel of all these polymerases, but once inside the enzyme, the GCSL seems to become stuck. This suggests that certain stable RNA motifs may have shapes or characteristics that lodges them unproductively inside the enzyme in a conformation that obstructs or inhibits processive translocation, and thereby causes stops. Previous single molecule studies with retroviral RTs have shown that, upon encountering template structures, polymerization is dominated by frequent kinetic pauses (1,3), and pause density is correlated with the relative base pair strength ahead of the polymerizing enzymes (3). These observations have led to the suggestion that retroviral RTs operate by a passive process that exploits the spontaneous thermal fluctuations of base pairs within template structures (3,28). This conclusion agrees well with our observation that SSIV is almost completely blocked by the GCSL (Figure 4D). The fact that MRT passes rapidly through all of the ‘challenge’ structures engineered in this study, and that only a few percent of molecules are blocked by the GCSL, suggests that group II intron RTs have evolved a different, active mechanism for smoothing out base pairs within template structures.

### Marching in unison: the implications of synchronized primer extension

One of the most striking features of the data obtained in this study is the remarkably synchronized pattern of primer extension by group II intron RTs, despite the heterogenous sequence content of the template. Previous studies using retroviral HIV RT and R2 RT from *Bombyx mori* R2 element have only demonstrated this type of processive, synchronized primer extension on homopolymeric poly(rA) templates and, even then, observed speeds were not uniform, ranging from 10–15 nt/s and 9.5–19.5 nt/s, respectively (4,29). In fact, when mixed sequence RNA templates were used, no synchronized pattern of extension was observed for either enzyme. It has never been clear how a population of RT enzyme behaves after it passes through an obstacle in the template, and whether this impacts the conformational ensemble of a population of translocating enzymes.

Our engineered ‘obstacle course,’ combined with our ability to visualize the speed and synchrony of an RT population at all positions, resulted in a methodology unique amongst studies of polymerases, and as such, it provided fresh insights into response of polymerases before, during and after encounters with template secondary and tertiary structure. For example, when monitoring the extending cDNA population that makes it past the GCSL structure, we observe the same synchronized pattern of extension and constant speed as seen in the original template, which has never been observed for any enzyme. Single molecule studies suggest that retroviral enzymes slow down upon encountering structures (3), but the use of short templates never allowed for evaluation of speed after encountering the structure. The homogeneity of MRT behavior, even after passing through stable structures, suggests that it does not sample any rate-limiting conformational states as it traverses obstacles and that the majority population of enzymes maintains a stable, single conformation during all stages of elongation. It is interesting to consider what actually controls the behavior of MRT, and the physical basis for its rate-limiting step. Given its insensitivity to stable structures in its path, template smoothing and unwinding are clearly not rate-limiting, so the inherent speed of MRT is likely due to intrinsic characteristics of the enzyme, such as the efficiency of nucleotide binding, product release or translocation along the RNA lattice. Determining the rate-limiting step for this remarkable enzyme family, and the physical basis for differences in velocity among polymerases will be an important subject for future

## CONCLUSION

This study has important practical ramifications for choosing an RT enzyme that is best-suited for certain types of transcriptome-wide analysis. Conventional retroviral RT enzymes severely limit the number and quality of reads that can be obtained on many types of natural RNA templates, therefore biasing the resultant transcriptomic datasets. Application of template-insensitive, processive group II intron RTs will enable researchers to capture the full spectrum of RNA transcript abundance, providing a wealth of information on novel gene targets and, potentially, leading to the discovery of new cellular RNA molecules. In addition to practical insights, this study underscores the microscopic diversity of RT behavior, demonstrating that each type of RT enzyme displays a unique pattern of behavior that arises from its specific role in biology and which lends itself to different types of technological applications. Despite the simplicity of their single-chain structures, we show that RTs like MRT are fast, processive, powerful enzymes that are largely insensitive to environmental conditions. The creative exploitation of these properties will lead to new insights into biological mechanism and new tools for exploring the expanding universe of functional RNA molecules.

## DATA AVAILABILITY

The sequencing data have been deposited to GEO under accession number GSE195979.

## Supplementary Material

gkac518_Supplemental_File

## References

[1] Klarmann G.J. , Schauber C.A., Preston B.D. Template-directed pausing of DNA synthesis by HIV-1 reverse transcriptase during polymerization of HIV-1 sequences in vitro. J. Biol. Chem. 1993; 268:9793–9802. 7683663

[2] Harrison G.P. , Mayo M.S., Hunter E., Lever A.M. Pausing of reverse transcriptase on retroviral RNA templates is influenced by secondary structures both 5' and 3' of the catalytic site. Nucleic Acids Res. 1998; 26:3433–3442. 9649630 10.1093/nar/26.14.3433 PMC147721

[3] Malik O. , Khamis H., Rudnizky S., Marx A., Kaplan A. Pausing kinetics dominates strand-displacement polymerization by reverse transcriptase. Nucleic Acids Res. 2017; 45:10190–10205. 28973474 10.1093/nar/gkx720 PMC5737391

[4] Bibillo A. , Eickbush T.H. High processivity of the reverse transcriptase from a non-long terminal repeat retrotransposon. J. Biol. Chem. 2002; 277:34836–34845. 12101182 10.1074/jbc.M204345200

[5] Zhao C. , Pyle A.M. Crystal structures of a group II intron maturase reveal a missing link in spliceosome evolution. Nat. Struct. Mol. Biol. 2016; 23:558–565. 27136328 10.1038/nsmb.3224 PMC4899126

[6] Cousineau B. , Smith D., Lawrence-Cavanagh S., Mueller J.E., Yang J., Mills D., Manias D., Dunny G., Lambowitz A.M., Belfort M. Retrohoming of a bacterial group II intron: mobility via complete reverse splicing, independent of homologous DNA recombination. Cell. 1998; 94:451–462. 9727488 10.1016/s0092-8674(00)81586-x

[7] Zhao C. , Rajashankar K.R., Marcia M., Pyle A.M. Crystal structure of group II intron domain 1 reveals a template for RNA assembly. Nat. Chem. Biol. 2015; 11:967–972. 26502156 10.1038/nchembio.1949 PMC4651773

[8] Qu G. , Kaushal P.S., Wang J., Shigematsu H., Piazza C.L., Agrawal R.K., Belfort M., Wang H.W. Structure of a group II intron in complex with its reverse transcriptase. Nat. Struct. Mol. Biol. 2016; 23:549–557. 27136327 10.1038/nsmb.3220 PMC4899178

[9] Stamos J.L. , Lentzsch A.M., Lambowitz A.M. Structure of a thermostable group II intron reverse transcriptase with template-primer and its functional and evolutionary implications. Mol. cell. 2017; 68:926–939. 29153391 10.1016/j.molcel.2017.10.024 PMC5728383

[10] Zhao C. , Liu F., Pyle A.M. An ultraprocessive, accurate reverse transcriptase encoded by a metazoan group II intron. RNA. 2018; 24:183–195. 29109157 10.1261/rna.063479.117 PMC5769746

[11] Xiong Y. , Eickbush T.H. Origin and evolution of retroelements based upon their reverse transcriptase sequences. EMBO J. 1990; 9:3353–3362. 1698615 10.1002/j.1460-2075.1990.tb07536.x PMC552073

[12] Guo L.T. , Adams R.L., Wan H., Huston N.C., Potapova O., Olson S., Gallardo C.M., Graveley B.R., Torbett B.E., Pyle A.M. Sequencing and structure probing of long RNAs using MarathonRT: a next-generation reverse transcriptase. J. Mol. Biol. 2020; 432:3338–3352. 32259542 10.1016/j.jmb.2020.03.022 PMC7556701

[13] Mohr S. , Ghanem E., Smith W., Sheeter D., Qin Y., King O., Polioudakis D., Iyer V.R., Hunicke-Smith S., Swamy S. et al. Thermostable group II intron reverse transcriptase fusion proteins and their use in cDNA synthesis and next-generation RNA sequencing. RNA. 2013; 19:958–970. 23697550 10.1261/rna.039743.113 PMC3683930

[14] Somarowthu S. , Legiewicz M., Chillon I., Marcia M., Liu F., Pyle A.M. HOTAIR forms an intricate and modular secondary structure. Mol. Cell. 2015; 58:353–361. 25866246 10.1016/j.molcel.2015.03.006 PMC4406478

[15] Wick R.R. , Judd L.M., Holt K.E. Performance of neural network basecalling tools for Oxford Nanopore sequencing. Genome. Biol. 2019; 20:129. 31234903 10.1186/s13059-019-1727-y PMC6591954

[16] Sedlazeck F.J. , Rescheneder P., Smolka M., Fang H., Nattestad M., von Haeseler A., Schatz M.C. Accurate detection of complex structural variations using single-molecule sequencing. Nat. Methods. 2018; 15:461–468. 29713083 10.1038/s41592-018-0001-7 PMC5990442

[17] Sexton A.N. , Wang P.Y., Rutenberg-Schoenberg M., Simon M.D. Interpreting Reverse Transcriptase Termination and Mutation Events for Greater Insight into the Chemical Probing of RNA. Biochemistry. 2017; 56:4713–4721. 28820243 10.1021/acs.biochem.7b00323 PMC5648349

[18] Schwartz J.J. , Quake S.R. Single molecule measurement of the ‘speed limit’ of DNA polymerase. Proc. Natl. Acad. Sci. U.S.A. 2009; 106:20294–20299. 19906998 10.1073/pnas.0907404106 PMC2787106

[19] Kelleher C.D. , Champoux J.J. Characterization of RNA strand displacement synthesis by Moloney murine leukemia virus reverse transcriptase. J. Biol. Chem. 1998; 273:9976–9986. 9545343 10.1074/jbc.273.16.9976

[20] Pop M.P. , Biebricher C.K. Kinetic analysis of pausing and fidelity of human immunodeficiency virus type 1 reverse transcription. Biochemistry. 1996; 35:5054–5062. 8664298 10.1021/bi9530292

[21] Chen X. , Chamorro M., Lee S.I., Shen L.X., Hines J.V., Tinoco I., Varmus H.E Structural and functional studies of retroviral RNA pseudoknots involved in ribosomal frameshifting: nucleotides at the junction of the two stems are important for efficient ribosomal frameshifting. EMBO J. 1995; 14:842–852. 7882986 10.1002/j.1460-2075.1995.tb07062.x PMC398151

[22] Toor N. , Keating K.S., Taylor S.D., Pyle A.M. Crystal structure of a self-spliced group II intron. Science. 2008; 320:77–82. 18388288 10.1126/science.1153803 PMC4406475

[23] Gerard G.F. , Potter R.J., Smith M.D., Rosenthal K., Dhariwal G., Lee J., Chatterjee D.K. The role of template-primer in protection of reverse transcriptase from thermal inactivation. Nucleic Acids Res. 2002; 30:3118–3129. 12136094 10.1093/nar/gkf417 PMC135738

[24] Gascon I. , Lazaro J.M., Salas M. Differential functional behavior of viral phi29, Nf and GA-1 SSB proteins. Nucleic Acids Res. 2000; 28:2034–2042. 10773070 10.1093/nar/28.10.2034 PMC105360

[25] Rodriguez I. , Lazaro J.M., Blanco L., Kamtekar S., Berman A.J., Wang J., Steitz T.A., Salas M., de Vega M. A specific subdomain in phi29 DNA polymerase confers both processivity and strand-displacement capacity. Proc. Natl. Acad. Sci. U.S.A. 2005; 102:6407–6412. 15845765 10.1073/pnas.0500597102 PMC1088371

[26] Brisco M.J. , Morley A.A. Quantification of RNA integrity and its use for measurement of transcript number. Nucleic Acids Res. 2012; 40:e144. 22735698 10.1093/nar/gks588 PMC3467039

[27] Suo Z. , Johnson K.A. Effect of RNA secondary structure on the kinetics of DNA synthesis catalyzed by HIV-1 reverse transcriptase. Biochemistry. 1997; 36:12459–12467. 9376350 10.1021/bi971217h

[28] Kim S. , Schroeder C.M., Xie X.S. Single-molecule study of DNA polymerization activity of HIV-1 reverse transcriptase on DNA templates. J. Mol. Biol. 2010; 395:995–1006. 19968999 10.1016/j.jmb.2009.11.072 PMC2818676

[29] Huber H.E. , McCoy J.M., Seehra J.S., Richardson C.C. Human immunodeficiency virus 1 reverse transcriptase. Template binding, processivity, strand displacement synthesis, and template switching. J. Biol. Chem. 1989; 264:4669–4678. 2466838

