# Supplementary material for: Direct tracking of reverse-transcriptase speed and template sensitivity: implications for sequencing and analysis of long RNA molecules
Source: Nucleic Acids Res. 2022 Jun 17;50(12):6980–9. doi: 10.1093/nar/gkac518 (PMC9262592; doi:10.1093/nar/gkac518)
Supplement: gkac518_Supplemental_File [file gkac518_supplemental_file.pdf]

Supplementary information

Direct tracking of reverse-transcriptase speed and template sensitivity: implications for sequencing and analysis of long RNA molecules

Li-Tao Guo[a], Sara Olson[e], Shivali Patel[d], Brenton R. Graveley[e] and Anna Marie Pyle[a,b,c,]*

[a] *Department of Molecular, Cellular, and Developmental Biology, Yale University, New Haven, Connecticut 06520, USA*

[b] *Howard Hughes Medical Institute, Chevy Chase, Maryland 20815, USA*

[c] *Department of Chemistry, Yale University, New Haven, Connecticut 06520, USA*

[d] *Department of Molecular Biophysics and Biochemistry, Yale University, New Haven, Connecticut 06520, USA*

[e] *Department of Genetics and Genome Sciences, Institute for Systems Genomics, UConn Health, Farmington, Connecticut 06030-6403, USA*

* To whom correspondence should be addressed. Email: anna.pyle@yale.edu

Suppl. Table 1. The DNA and RNA oligos used in this work.

DNA oligos	Sequence	Note
RT_speed	5'-TTCTGTTCTTCTGTAC TTCTA-3'	Primer for ^{32}P labeling to determine primer extension speed
RT_speed_FAM	5'-FAM-TTCTGTTCTTCT GTACTTCTA-3'	FAM labeled primer to determine primer extension speed
RTstop_primer1	5'-CAGACGTGTGCTCTT CCGATCTGAGCTTGGG TGTAATTGCTGG -3'	One of the four primers used to reverse transcribe HOTAIR templates (WT and GCSL) for RT stop profiling
RTstop_primer2	5'-CAGACGTGTGCTCTT CCGATCTCCAGCTCTC TGGTCTTGTTAAC-3'	The second primer used to reverse transcribe HOTAIR templates (WT and GCSL) for RT stop profiling
RTstop_primer3	5'-CAGACGTGTGCTCTT CCGATCTGTCTAGGAAT CAGCACGAAGC-3'	The third primer used to reverse transcribe HOTAIR templates (WT and GCSL) for RT stop profiling
RTstop_primer4	5'-AGACGTGTGCTCTTC CGATCTTTCT GTTCTTC TGTACTTCTA-3'	The fourth primer used to reverse transcribe HOTAIR templates (WT and GCSL) for RT stop profiling
RTstop_3adapter	5'-p-NNNNNNAGATCGG AAGAGCGTC GTGTAG-biotin-3'	The ssDNA adapter for ligation to the 3'-end of cDNAs to construct sequencing libraries
SC_trap	5'-GGGGATCCTAATACGAC TCACTATAGGGATGCATGT AGACACAGAAGGGGddC-3'	ssDNA trap tested in single-cycle reverse transcription
**RNA oligos**		
RNA1	5'-CAGAAGGGGUAUUUAAA AGACAGAAAUAAUAGAAG U ACAGAAGAACAGAA-3'	RNA trap tested in single-cycle reverse transcription
RNA2	5'-GCAACCACGAAGCUAGA GAGAGAGCCAGAGGAGGG AAGAGAGCGCCAGAC-3'	RNA trap tested in single-cycle reverse transcription

Suppl. Table 2.
The nucleotide sequences of the RNA structures inserted into HOTAIR RNA

12-bp GC stem loop	GUUAAUAACGGGCCCGCCGGCUUUUGCCGGCGGGCCCUU<br>UUAUUU
Mouse mammary tumor virus (MMTV) gag-pro frameshift site RNA pseudoknot	UUCGAAAGGGGCAGUCCCCUAGCCCCACUCAAAAGGGGGA<br>U AAAGGUA
E. coli $tRNA^{Phe}$	AAUAUCAGUUGCCCGGAUAGCUCAGUCGGUAGAGCAGGG<br>GAUUGAAAAUCCCCGUGUCCUUGGUUCGAUUCCGAGUCC<br>GGGCACCAAAUUCAUAUA
Oceanobacillus iheyensis group II intron	UUUUUUUAUGUGUGCCCGGCAUGGGUGCAGUCUAUAGGG<br>UGAGAGUCCCGAACUGUGAAGGCAGAAGUAACAGUUAGCC<br>UAACGCAAGGGUGUCCGUGGCGACAUGGAAUCUGAAGGA<br>AGCGGACGGCAAACCUUCGGUCUGAGGAACACGAACUUCA<br>UAUGAGGCUAGGUAUCAAUGGAUGAGUUUGCAUAACAAAA<br>CAAAGUCCUUUCUGCCAAAGUTUGUACAGAGUAAAUGAAG<br>CAGAUUGAUGAAGGGAAAGACUGCAUUCUUACCCGGGGAG<br>GUCUGAUCGAAACGCCAAGCACUCUUGGUAACCCAUUCAG<br>CAAUGGAUGGCUGAACGGUCAGAAGUCAGCAGAAGUCAUA<br>GUACCCUGCAUACUCGAGAAUGUAAGGGGAAGGACGGAAC<br>AAUUAAGUUCGCUUAAUUGAACCGCCGUATACCGAACGGU<br>ACGUACGGUGGUGUGAGAGGACGGGGGUUAGUCGCUCCC<br>UUCUACUCUAUUAUUUAUCA

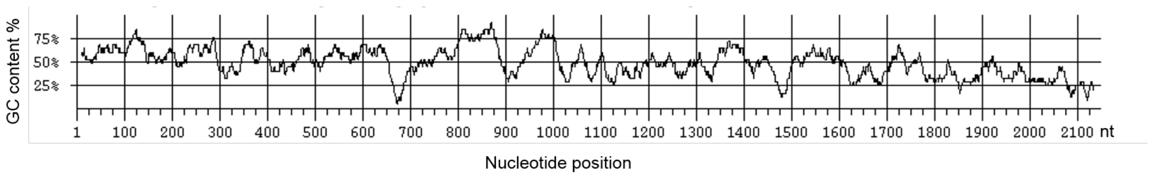

Supplementary Figure 1. Scan of GC content along the 2148-nt HOTAIR RNA using a window size of 25 bp.

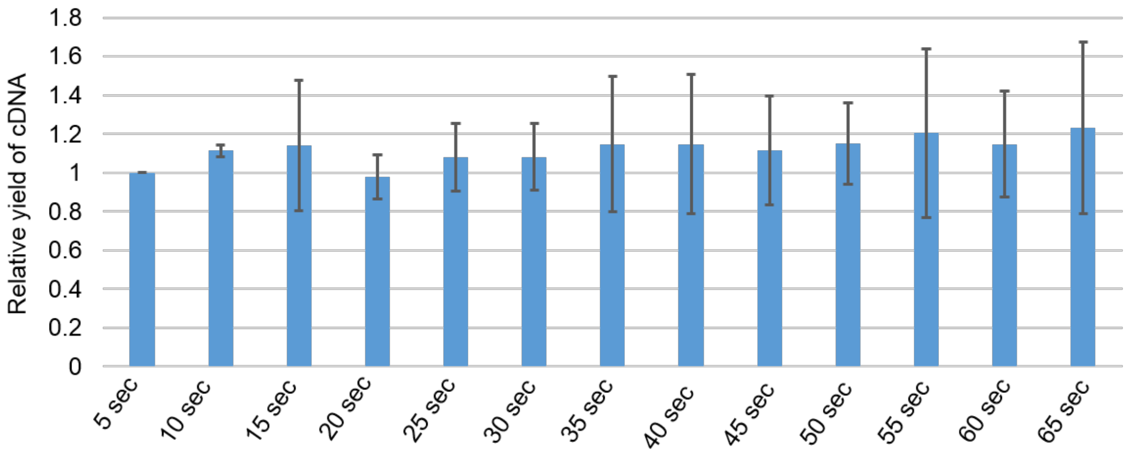

Supplementary Figure 2. Relative yield of cDNA products generated by the population of synchronized MarathonRT at each reaction time. The cDNA products represented by the front of extended primers, as shown in **Fig. 1B,** was quantified at each reaction time. The amount observed at each reaction time was normalized to that observed at 5 sec, which was set as 1. The quantification was generated from 3 replicates. The error bar for each time point represents the standard deviation.

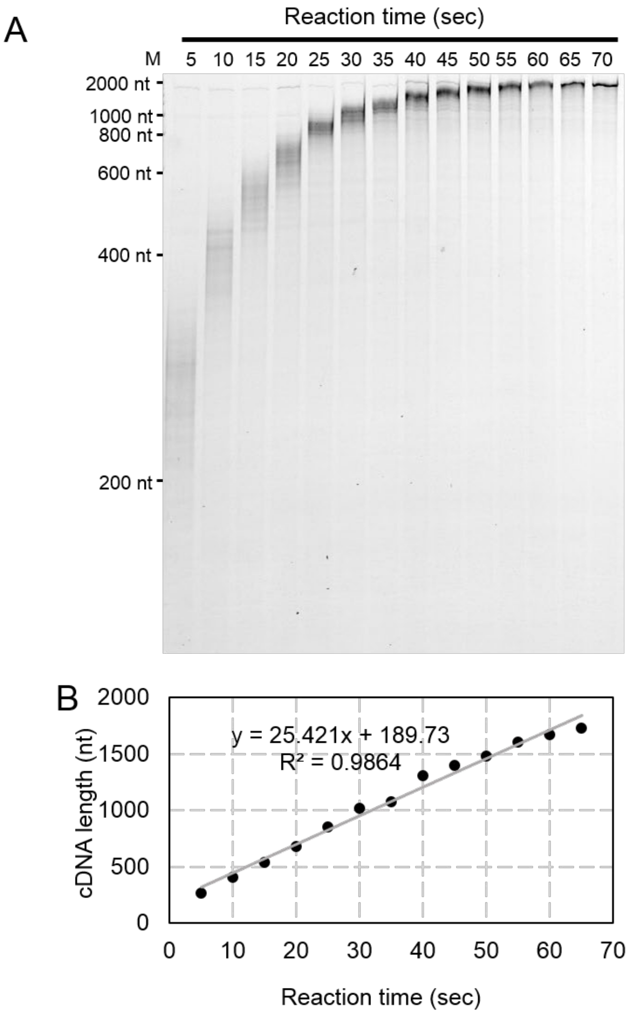

Supplementary Figure 3. Monitoring the primer extension progress for MarathonRT with a FAM labeled primer. HOTAIR RNA was used as the template. (A) Analyzing primer extension at the indicated reaction times on a denaturing polyacrylamide gel via FAM labeling. The FAM labeled DNA size marker (M) was used to determine the length of the cDNA products. (B) Plot of the average length of the cDNA products vs reaction time. The velocity was calculated to be 25.4 nt/sec.

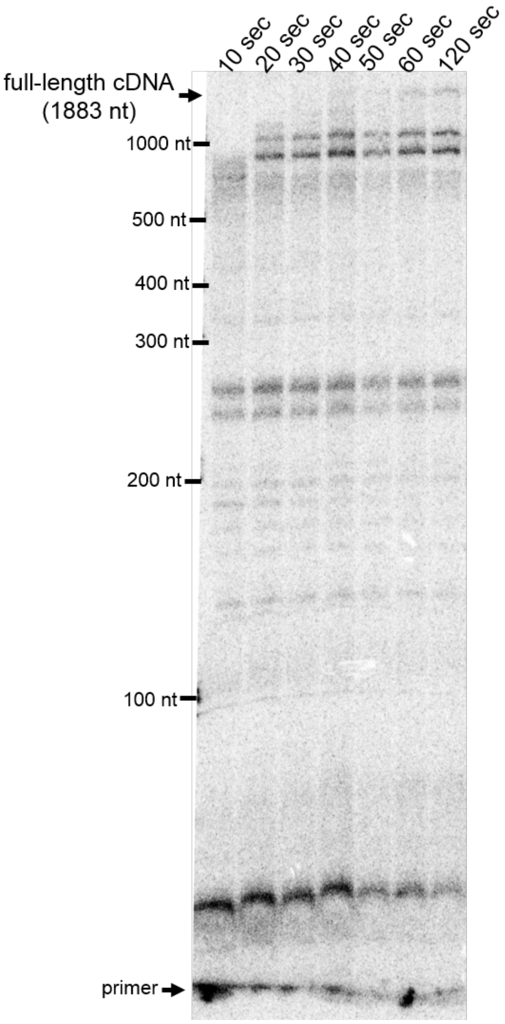

Supplementary Figure 4. Examining the time-resolved primer extension by SuperScript IV on HOTAIR RNA template. The cDNA products from each reaction time were analyzed on a denaturing polyacrylamide gel and visualized via ^{32}P labeling.

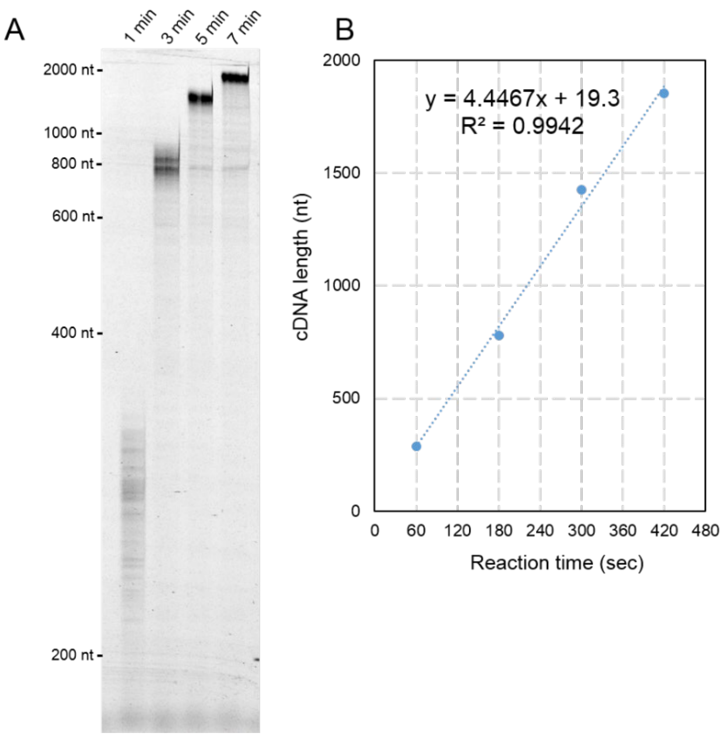

Supplementary Figure 5. Determining the velocity of TGIRT III. (A) Analyzing primer extension on HOTAIR RNA template at the indicated reaction times on a denaturing polyacrylamide gel via FAM labeling. (B) Plot of the average length of cDNA products vs. reaction time. The velocity was calculated to be 4.45 nt/sec.

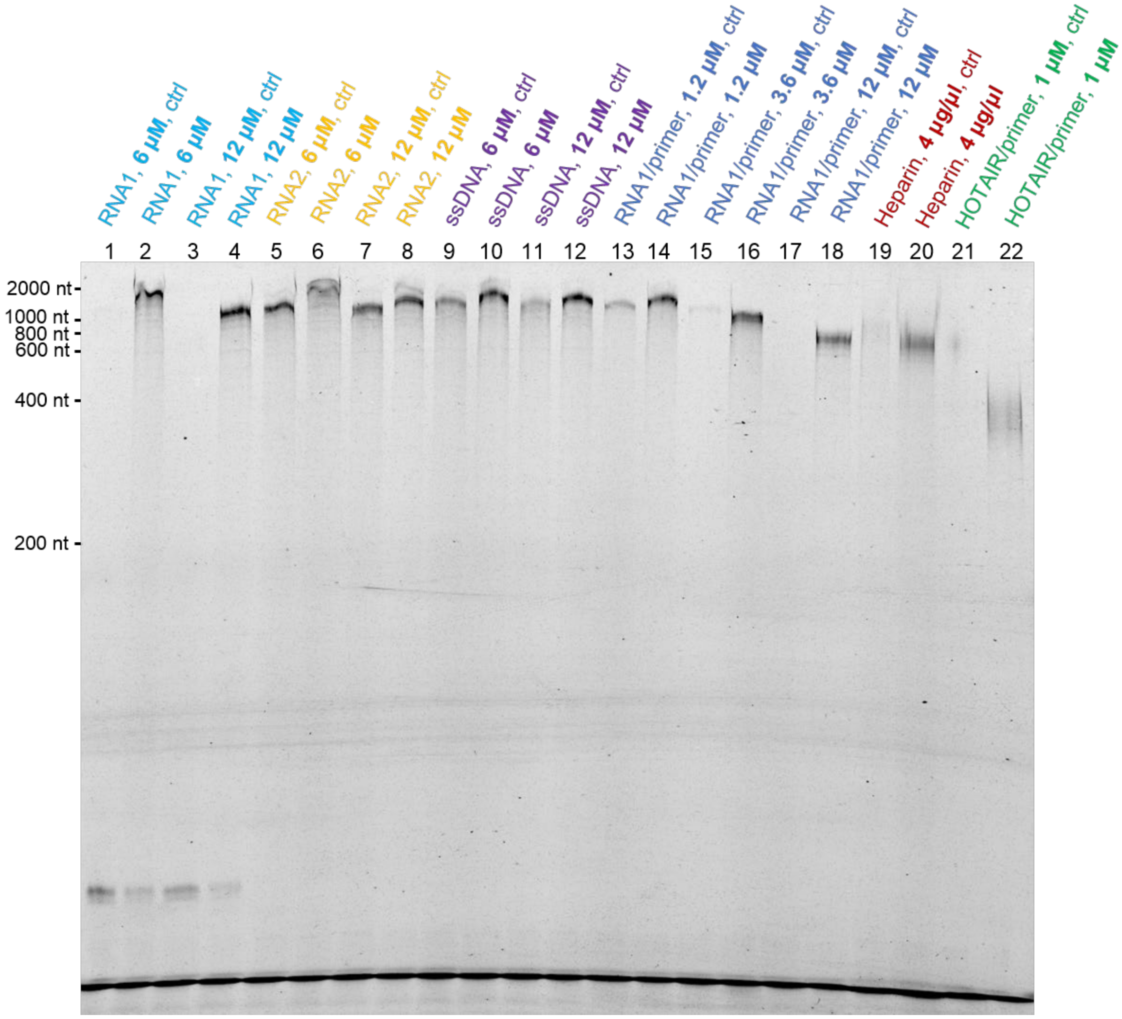

Supplementary Figure 6. Single-cycle reverse transcription by MarathonRT on HOTAIR RNA template. In these experiments, any dissociated RT was trapped by the addition of a trap molecule (see Materials and Methods) when initiating the reaction. In the control group (Lane 1, 3, 5, 7, 9, 11, 13, 15, 17, 19 and 21), MarathonRT was preincubated with each of the traps before being presented to the annealed template-primer. The traps used in this assay include two 50-nt single-stranded RNAs (RNA1, Lane 1 – 4, and RNA2, Lane 5 – 8), one 50-nt single-stranded DNA (ssDNA, Lane 9 – 12), RNA1 annealed with an unlabeled RT primer (RNA1/primer, Lane 13 – 18), Heparin (Lane 19 – 20) and HOTAIR RNA annealed with an unlabeled primer (HOTAIR/primer, Lane 21 – 22). Their concentrations are described in the figure. All the reactions were incubated for 1 min at 42℃. Only RNA1 at 6 μM is effective in preventing dissociated MarathonRT from rebinding to the template (Lane 1) without affecting MarathonRT ability to produce full length cDNA product in 1 min (Lane 2).

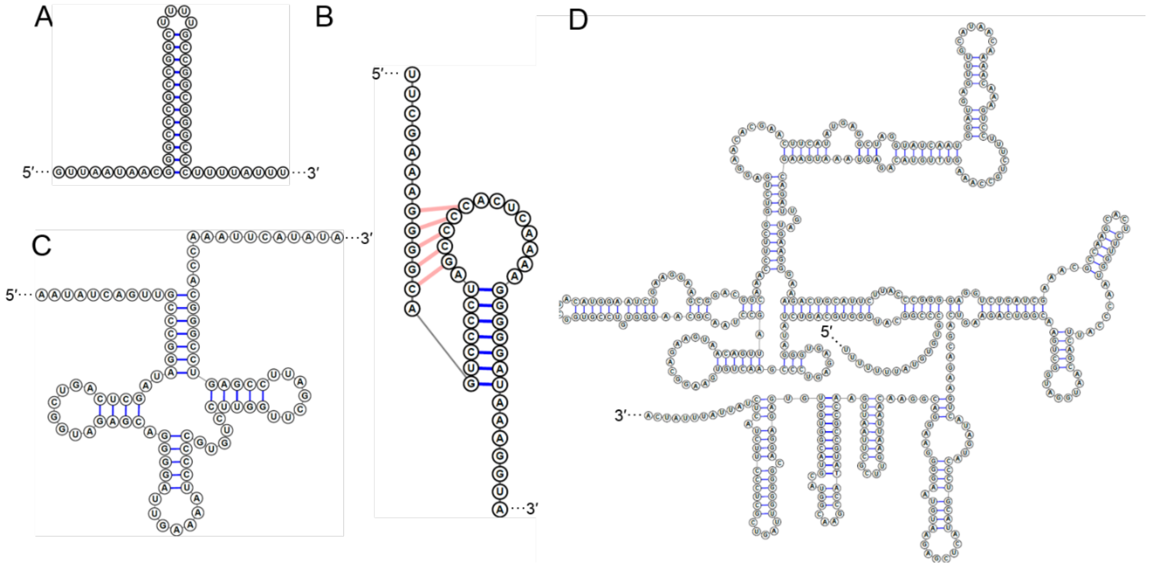

Supplementary Figure 7. Four RNA structures that were inserted into the HOTAIR template. (A) GC stem loop. (B) MMTV pseudoknot. (C) *E. coli* tRNA^{Phe} (D) *O.i.* group II intron.

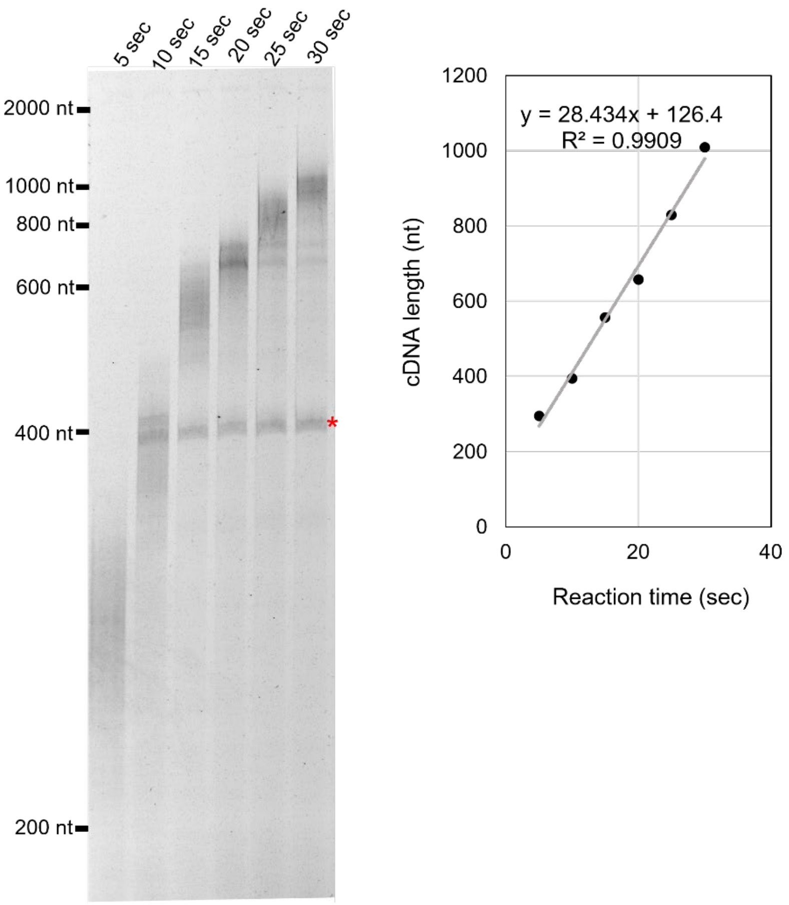

Supplementary Figure 8. Time course experiment to monitor the primer extension progress of MarathonRT on the HOTAIR GCSL template. (A) Electrophoresis analysis of the cDNA products generated at different reaction times as denoted in the legend. The cDNA products were visualized via FAM labeling on a denaturing polyacrylamide gel. The GCSL induced stop was denoted by red asterisk. (B) Plot of the average length of cDNA products against reaction time. The velocity was calculated to be 28.4 nt/sec.

Supplementary Python scripts

File name: LRlengthDistribution.py

The Python script was used to calculate the length distribution of long reads from nanopore sequencing. The sequencing reads were produced from cDNA generated by MarathonRT, and the length distribution datasets calculated with this Python script were plotted to generate Fig. 2A.

Requirement: Python 2.7

The Python scripts requires BED files as the input. To obtain the BED files, the sequencing reads were aligned to the HOTAIR template by ngmlr 0.2.7 (see MATERIALS AND METHODS for details) to obtain SAM files, which were then converted to BAM files by SAMtools. The BAM files were used to generate the BED files by BEDTools.

Usage:

1. Replace the strings in “sample_list” with your own sample names with suffix excluded.
2. Copy the Python script below to create the file LRlengthDistribution.py.
3. Run the script in the form of:

   python2 LRlengthDistribution.py /home/path/to/BED/ /home/path/to/output/

```
################################################################################

import sys
import math
import fileinput
import shutil
import time
import os         #module for various functions for interacting with the operating system
import shlex      #module for functions...
import subprocess  #module facilitates interactions with other shells, for example call bowtie...

args_in = list(sys.argv)
#directory to the BED files (/home/path/to/BED/)
bed_folder=args_in[1]
#directory for the output AND txt files summarizing your results  (/home/path/to/output/)
output_folder=args_in[2]

sample_list = ['sample1', 'sample2', 'sample3', '…']

distribution = open(output_folder+'Hotair12_PrimerBind_LenDist50.txt', 'w')
line = 'length bin:\t'
distribution.write(line)
binz = []
for w in range(0,2200, 50):
    bin1 = str(w)
```

```
        binz.append(bin1)
    line = "\t".join(binz)
    distribution.write(line)
    line = '\n'
    distribution.write(line)

    dist_dict = {}
    for sample in sample_list:
        sample_name = sample
        print(sample_name)
        bed_file = open(bed_folder+sample_name+'.bed', 'r')

        primer_bind = 0
        dist_dict[sample_name] = []
        len_distribution = {}
        for w in range(0,2200, 50):
            len_distribution[w] = 0

        len_sum = 0

        for line in bed_file:
            columns = line.rstrip('\n').split('\t')
            start = int(columns[1])
            end = int(columns[2])
            if end >= 1820:
                primer_bind+=1
                seq_length = int(end - start)
                len_sum = len_sum+seq_length
                for key in len_distribution:
                    left = key
                    right = key + 49
                    if seq_length >= left and seq_length <= right:
                        count = len_distribution[key]
                        count+=1
                        len_distribution[key] = count
                    else:
                        pass
                else:
                    pass
            else:
                pass

        bed_file.close()
        average_len = len_sum/primer_bind

        print('primer binding:')
        print(primer_bind)
        print('average length of primer bound sequences: ')
```

```
        print(average_len)

        for w in range(0,2200, 50):
                count = len_distribution[w]
                count = str(count)
                dist_dict[sample_name].append(count)

for i in range(0,len(sample_list),1):
        name = sample_list[i]
        distribution.write(name+'\t')
        count_list = dist_dict[name]
        line = "\t".join(count_list)
        distribution.write(line)
        line = "\n"
        distribution.write(line)

print ('done...')
```
